# Identification and validation of molecular subtypes and prognostic signature for stage I and stage II gastric cancer based on neutrophil extracellular traps

**DOI:** 10.1515/med-2023-0860

**Published:** 2024-01-12

**Authors:** Lei Mu, Gang Qiu

**Affiliations:** Emergency Surgery, Sunshine Union Hospital, 9000 Yingqian Road, High-tech Zone, Weifang, Shandong, 261000, China

**Keywords:** stage I and stage II gastric cancer, neutrophil extracellular traps, molecular subtype, prognostic signature

## Abstract

**Purpose:**

This study identified subtypes and prognostic signature of stage I and stage II gastric cancer based on neutrophil extracellular trap (NET)-related genes.

**Methods:**

The gene expression data associated with stage I and stage II gastric cancer were downloaded from The Cancer Genome Atlas (TCGA) and Gene Expression Omnibus (GEO) databases. NET-related genes were obtained from previous reference. Differentially expressed NET-related genes were selected by consensus cluster analysis. The differences in immune infiltration between two subtypes were analyzed. Prognosis-related genes were further screened by univariate Cox regression analysis. Gene Set Enrichment Analysis (GSEA) of prognostic signatures was conducted with clusterprofiler. Finally, a miRNA–mRNA–transcription factor (TF) network was constructed.

**Results:**

Total 43 differential NET-related genes were obtained and two subtypes were obtained based on these genes. Patients of cluster 2 had a better prognosis compared to cluster 1. Eight types of immune cells were differential in infiltration level between two subtypes. Following univariate Cox regression analysis, two genes of CXC chemokine receptor 4 (*CXCR4*) and nuclear factor, erythroid 2-like 2 (*NFE2L2*) significantly related to patient survival were selected. GSEA of single gene revealed that *CXCR4* was associated with allograft rejection and *NFE2L2* was associated with drug metabolism-cytochrome P450. A network with 421 miRNA–mRNA–TF regulatory pairs was constructed.

**Conclusion:**

The present study identified two subtypes and a prognostic signature for stage I and stage II gastric cancer based on NET-related genes.

## 1 Introduction

Gastric cancer was the fifth most common malignancy as reported in 2018 [1]. Despite the decline in incidence, gastric cancer remains the fifth leading cause of cancer-related death, accounting for 8.2% of all cancer deaths worldwide [2]. Stage I and stage II gastric cancer has a favorable prognosis, with the 5-year survival rate of 69–82%, indicating the importance of early diagnosis and treatment [3]. Regarding the relatively good prognosis, further postoperative review and follow-up are usually omitted for stage I and stage II gastric cancer. The outcomes of gastric cancer may vary significantly in patients with the similar therapy management [4]. An effective detection method for stage I and stage II gastric cancer requires the combination of initial preselection and follow-up endoscopy in high-risk populations, but its large-scale application faces challenges [5]. Therefore, there is an urgent need for a rapid, non-invasive method to predict the recurrence risk of stage I and stage II gastric cancer and improve prognosis.

Neutrophil is the most abundant leukocyte among peripheral blood and can fight off inflammatory or infectious insults [6]. Neutrophils are antimicrobial active through phagocytosis, pelleting of cytotoxic enzymes and proteases, and neutrophil extracellular traps (NETs) [7]. NET is described as an extracellular fiber responsible for capturing and killing the DNA and modifying proteins of extracellular pathogens [8]. NETs play various roles in cancer-related inflammation, such as promoting tumor growth and distant metastasis [9]. It has been reported that levels of circulating NETs are increased in patients with advanced gastric cancers in comparison with local cancers and healthy person [10], and NETs formed *in situ* rather than in peripheral blood might be a more direct prognostic indicator in tumor patients. However, NET-related genes (NRGs) and their prognostic value and relationship with immune microenvironment in stage I and stage II gastric cancer remain unclear.

In this study, based on several public databases, we first analyzed the NET-related molecular subtypes for stage I and stage II gastric cancer. Then a prognostic signature related to NETs was constructed by univariate Cox regression analysis. Additionally, the regulatory network associated with the NRGs was constructed to explore the potential regulatory mechanism. This study may provide new evidence about the key role of NETs in the prognosis of patients with stage I and stage II gastric cancer.

## 2 Methods

### 2.1 Data sources

RNA-seq data, survival, and clinical information of patients with gastric cancer (TCGA-STAD, The Cancer Genome Atlas-gastric adenocarcinoma) in UCSC (University of California Santa Cruz) database were used. According to the clinical information (tumor stage), samples of gastric cancer (stage I and stage II) were selected. A total of 164 samples of gastric cancer and 32 normal samples were included in the RNA-seq expression matrix, and 156 cancer samples had both survival and clinical information. The clinical characteristics of tumor patients and normal controls are listed in Table S1.

Additionally, gastric cancer dataset GSE66229 was downloaded from the Gene expression omnibus (GEO) database, which was used to validate the expression level and prognosis value of biomarkers. Similarly, the samples of gastric cancer (stage I and stage II) were screened according to the sample information stage of dataset. The gene expression matrix of this dataset contained 127 samples of stage I and stage II gastric cancer and 100 normal control samples, and 127 cancer samples had survival information.

Moreover, 136 NRGs were obtained from previous reference [11].

### 2.2 Differentially expressed genes (DEGs) screening

With the gene expression matrix of TCGA-STAD samples obtained from the UCSC database, the DEGs between gastric cancer and normal samples were screened by R package limma 3.42.2 [12], and visualized by R package ggplot2 3.3.2 [13] and pheatmap 0.7.7 [14]. Limma is a kind of generalized linear model, which fits the expression of each gene into a linear equation. Limma model contained ANOVA, linear regression analysis, combined with continuous variables (such as gene expression dataset) and categorical variables (such as sample grouping). The Benjamini & Hochberg method was adopted to conduct multiple test correction. Adjusted *p* value <0.05 and |log2 fold change (FC)| >0.5 were used as thresholds.

### 2.3 Differentially expressed NRGs screening and function analysis

The obtained DEGs were intersected with NRGs using online tool jVenn [15]. The clustering heatmap of intersection genes between sample groups was drawn using pheatmap 0.7.7 and the location of intersection genes on chromosomes was visualized using R package RCircos 1.2.2 [16].

Gene ontology (GO) and Kyoto encyclopedia of genes and genomes (KEGG) analyses of intersection genes were conducted using R package clusterprofiler 3.14.3 [6], and the enrichment results were visualized using ggplot2 3.3.2.

### 2.4 Consensus cluster analysis

ConsensusClusterPlus 1.54.0 [17] was used for consensus cluster analysis of 164 samples of stage I and stage II gastric cancer in the TCGA dataset based on the differentially expressed NRGs obtained above. Clustering parameters were set as maxK = 6, clusterAlg = “pam,” and distance = “pearson.” Principal component analysis (PCA) was utilized to evaluate the sample clustering results. Then combined with the survival information of the samples, the R survival 3.2-13 [18] was used to perform Kaplan–Meier (KM) survival analysis for the different subtypes of samples.

### 2.5 Pathway analysis of different subtypes

The background gene set of c5.go.bp.v7.5.1.symbols.gmt was obtained from MSigDB database [19]. The enrichment score of each function was calculated using ssGSEA in R gene set variation analysis (GSVA 1.34.0) [20]. The differential functional pathways among different subtypes were screened out using limma, and the heatmap of differential functional pathways was displayed using pheatmap 0.7.7.

### 2.6 Analysis of immune cell infiltration

Infiltration of immune cell in different subtypes was analyzed through ssGSEA algorithm of GSVA 1.34.0. The immune cells with differential infiltration between subtypes were analyzed by wilcox.test method and displayed in boxplot produced using ggplot2 package in R.

### 2.7 Prognosis-related differentially expressed NRGs screening

Based on the expression of differentially expressed NRGs in TCGA cancer samples, combined with the survival and prognosis information of the samples, univariate Cox regression analysis was conducted using R survival 3.2-13 [18] to screen prognosis-related genes and *p* < 0.05 was used as the threshold.

### 2.8 Prognosis signature screening

Stage I and stage II gastric cancer samples were classified into the high- and low-expression groups according to the optimal threshold of prognosis-related gene above using the R package survminer 0.4.8 [21]. In combination with the survival information, KM survival analysis was performed on patients in the high- and low-expression groups using the survival package in R, and genes with survival significance were screened out as the prognostic biomarkers

### 2.9 Correlation between gene biomarker and clinical features

To understand the correlation between biomarkers and clinical features, the clinical information (age, gender, neoplasm histologic grade, pathologic T and N, tumor stage, overall survival [OS]) of the samples was extracted from the TCGA data set. In combination with different clinical features of patients, wilcox.test or Kruskal–Wallis test was used to analyze the correlation between each gene biomarker and clinical features. ROC curve was performed to compare the predictive accuracy of the two gene biomarkers in the 1-, 3-, 5-year survival rate of gastric cancer patients.

### 2.10 Gene set enrichment analysis (GSEA)

First, the Pearson correlation coefficients between each gene biomarker and the other genes in the TCGA dataset were calculated, respectively. Genes that significantly correlated with marker gene were collected based on the cutoff value of *p* <0.05. Following that, GO and KEGG enrichment analyses was conducted by GSEA algorithm using clusterprofiler 3.14.3. Benjamini & Hochberg method was adopted for multiple test correction, and adjusted *p* value <0.05 was considered as significant enrichment result.

### 2.11 Single nucleotide variation (SNV) and copy number variation (CNV) analysis

The SNV and CNV data of stage I and stage II gastric cancer were retrieved from TCGA database. Maftools package in R were implemented to analyze the SNV frequency of genes in gastric cancer patients and the CNV frequency of differentially expressed NETs.

### 2.12 Drug sensitivity analysis

According to Genomics of drug sensitivity in cancer cell line profile and TCGA-STAD gene expression profile, pRRophetic 0.5 [22] was used to construct Ridge regression model to predict IC50 of drugs based on the expression profiles of TCGA cancer samples. The Wilcox test was used to screen the drugs that showed significant differences in sensitivity between high- and low-expression groups of target genes.

### 2.13 Regulatory network analysis

MiRNAs targeting each gene biomarker was carried out using the miRWalk3.0 database, and the default parameters were adopted. Meanwhile, the predicted miRNAs were also retrieved from the miRDB database. Additionally, transcription factors (TFs) were predicted using the hTFtarget database [23].

With the obtained miRNA–mRNA and mRNA–TF relation pairs, the miRNAs and TFs that regulated the same mRNA were selected to establish the miRNA–mRNA–TF regulatory network using the Cytoscape 3.6.1 [24].

### 2.14 Validation of expression and prognostic value of gene signature

The expression levels of biomarkers were extracted from the TCGA dataset and the validation set (GSE66229), respectively. Ggplot2 3.3.2 software was used to plot the expression level of gene biomarkers in gastric cancer samples and normal controls. The difference of gene expression was analyzed by Wilcox test with the significance threshold value of *p* < 0.05.

Additionally, in the validation set (GSE66229), survminer 0.4.8 was used to divide gastric cancer samples into high- and low-expression group according to the optimal threshold of each gene signature. Combined with the survival information of patients, KM survival analysis was performed for patients in the high- and low-expression group using survival 3.2-13.

### 2.15 Cell culture and clinical sample collection

Gastric cancer cell lines AGS and MKN-45 as well as human normal gastric mucous epithelium cell GES-1 were obtained from the Chinese Academy of Sciences Cell Bank (Shanghai, China). All the cells were maintained in the RPMI-1640 culture medium containing 10% fetal bovine serum and penicillin/streptomycin at 37°C with 5% CO_2_.

Five paired gastric tumor samples and normal counterparts were collected from our hospital with fully informed consent. After approval by Ethics Committee of our hospital, the human tissues related study was performed according to the Declaration of Helsinki.

### 2.16 Real-time quantitative PCR (qPCR) analysis

The expressions of six genes of interest, including CXC chemokine receptor 4 (*CXCR4*), *SPP1*, *CXCL1*, *MMP9*, *TIMP1*, and nuclear factor, erythroid 2-like 2 (*NFE2L2*) in gastric cancer cells and tissue samples were detected using qRT-PCR. Briefly, total RNA was extracted from cells and tissues using TRIzol reagents. RNAs was reversely transcribed into cDNAs with the implement of PrimeScript™ RT reagent kit (Takara, Kusatsu, Japan) as per the manufactory’s recommendation. Then, PCR amplification was performed following the procedure of 94°C for 5 min, 30 cycles of 94°C for 45 s, 56°C for 30 s and 72°C for 30 s, and finally 72℃ for 10 min. The expressions of key genes were determined by qRT-PCR using ABI7500 system. The PCR primer sequences are listed in Table S2. Gene expression was normalized to GAPDH. RNA was quantified using 2^−ΔΔCt^ method.

### 2.17 Statistical analysis

Statistical analyses were conducted by using SPSS 19.0 (SPSS, Chicago, USA). Student’s *t*-test was utilized for comparison of difference between the two groups. *p*  <  0.05 was considered significant.

## 3 Results

### 3.1 DEGs analysis

**Figure 1:**
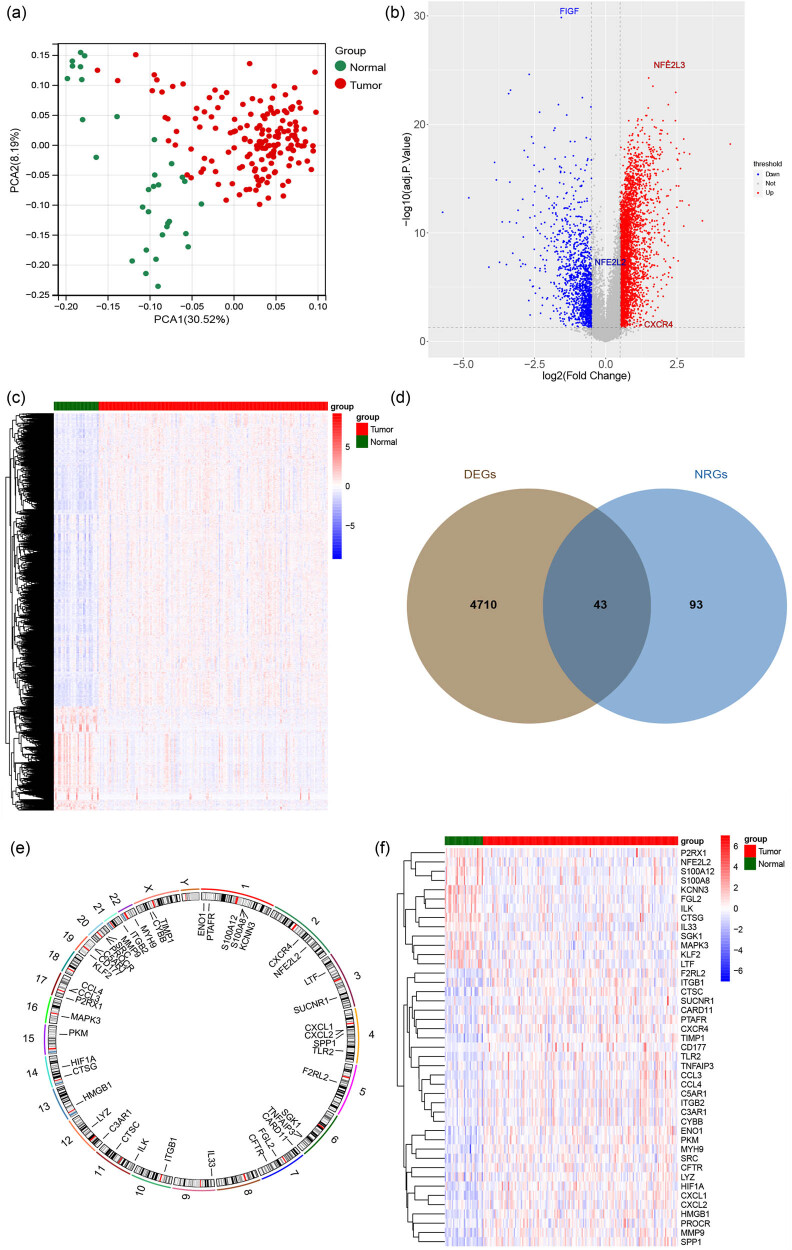
PCA and differentially expressed NRGs screening. (a) PCA analysis of gastric tumor samples and normal controls. (b) Volcano map of DEGs between sample groups (tumor vs normal). (Each dot represents a gene. The red dots indicate up-regulated genes, the blue dots indicate down-regulated genes, and the gray dots indicate no significant differences in these genes.) DEGs between tumor and normal samples were analyzed by limma package in R. (c) Heat map of DEGs between sample groups. (Each small square represents a gene, and its color indicates the gene expression level. The higher the expression level, the darker the color (red is high expression, blue is low expression.) (d) Intersection gene Venn diagram. (e) Loop graph of intersection genes at chromosomal locations. (f) Intersectional gene expression heat map between sample groups.

PCA was used to obtain the overall similarity between the samples. Figure 1a shows that the samples between normal and tumor groups were clearly distinguished, indicating the general different gene expression levels between the groups. Under adjusted *p* value <0.05 and |log2 FC| >0.5, 4,753 (3,512 up- and 1,241 down-regulated) DEGs were obtained in tumor versus normal groups. The volcano map was used to display the distribution of DEGs (Figure 1b), and the heatmap was used to present the expression of DEGs between the two groups (Figure 1c).

Moreover, the intersection of NRGs and the above DEGs was considered as differentially expressed NRGs, obtaining 43 intersection genes (Figure 1d). The location of intersection genes on chromosomes is presented in circus map (Figure 1e). The heatmap of intersection genes between sample groups is shown in Figure 1f.

### 3.2 Function analysis

**Figure 2:**
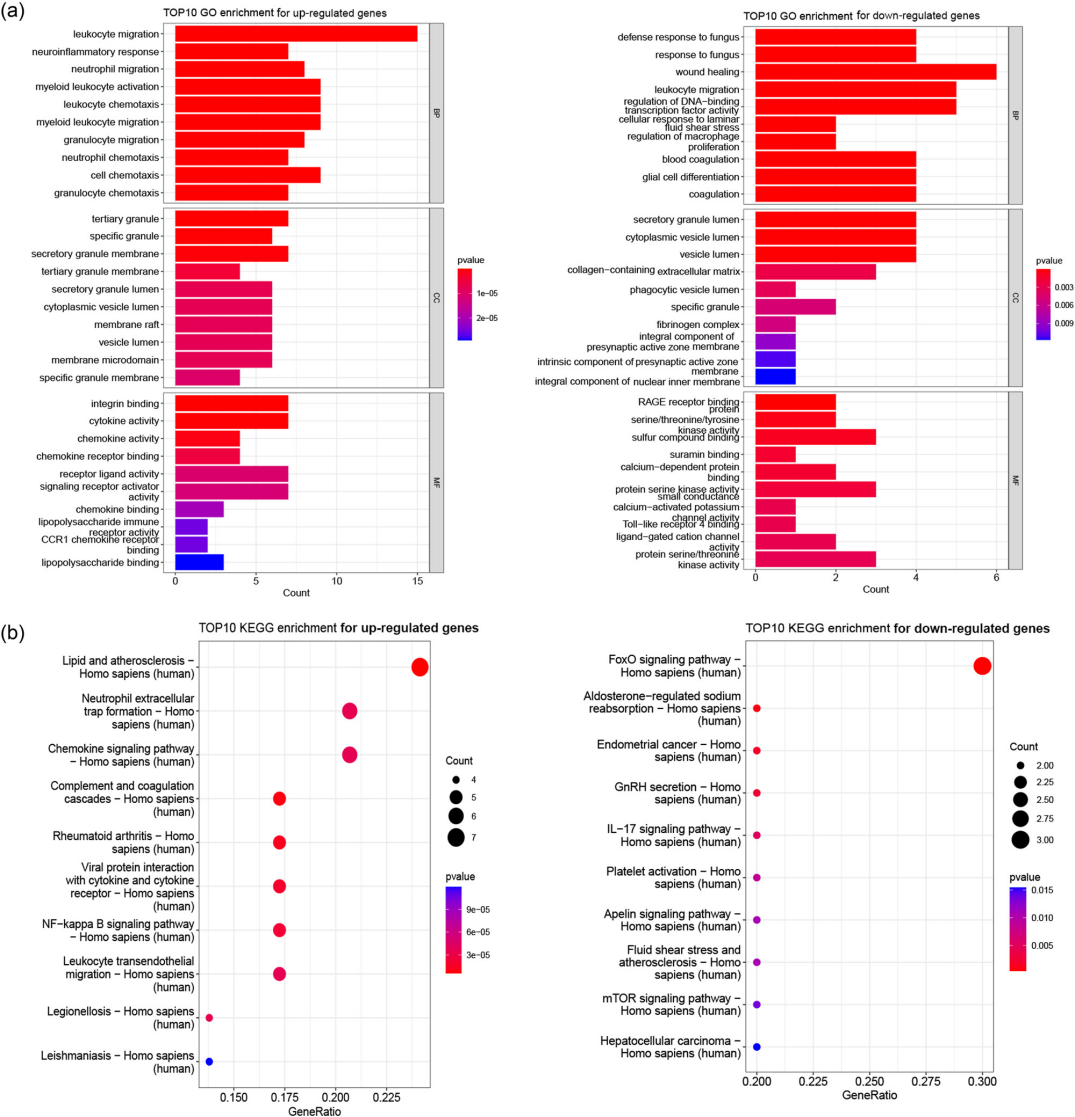
Function analysis of NRGs. (a) GO enrichment results (TOP10) bar graph. Left, up-regulated genes; right, down-regulated genes. (b) KEGG enrichment results (TOP10) bubble graph of intersection gene. Left, up-regulated genes; right, down-regulated genes.

To dissect the mechanism role of differentially expressed NRGs (30 up-regulated genes and 13 down-regulated genes), GO and pathway enrichment analysis were performed. Under adjusted *p* value <0.05, up-regulated genes were significantly enriched in 1,198 GO biological processes (BP), 82 cellular components (CC), 131 molecular function (MF) terms, and 48 KEGG pathways. The top ten BP terms included leukocyte migration, leukocyte chemotaxis, and granulocyte migration. (Figure 2a). The down-regulated genes were closely related with 582 GO-BP terms, 28 GO-CC terms, 53 GO-MF terms, and 20 KEGG pathways. The significant pathways involved with up-regulated genes were immune and inflammation related, such as chemokine signaling pathway, NET formation, and NF-kappa B signaling pathway. The down-regulated genes were significantly enriched in IL-17 signaling pathway, mTOR signaling pathway, and FoxO signaling pathway (Figure 2b).

### 3.3 Consensus clustering analysis

**Figure 3:**
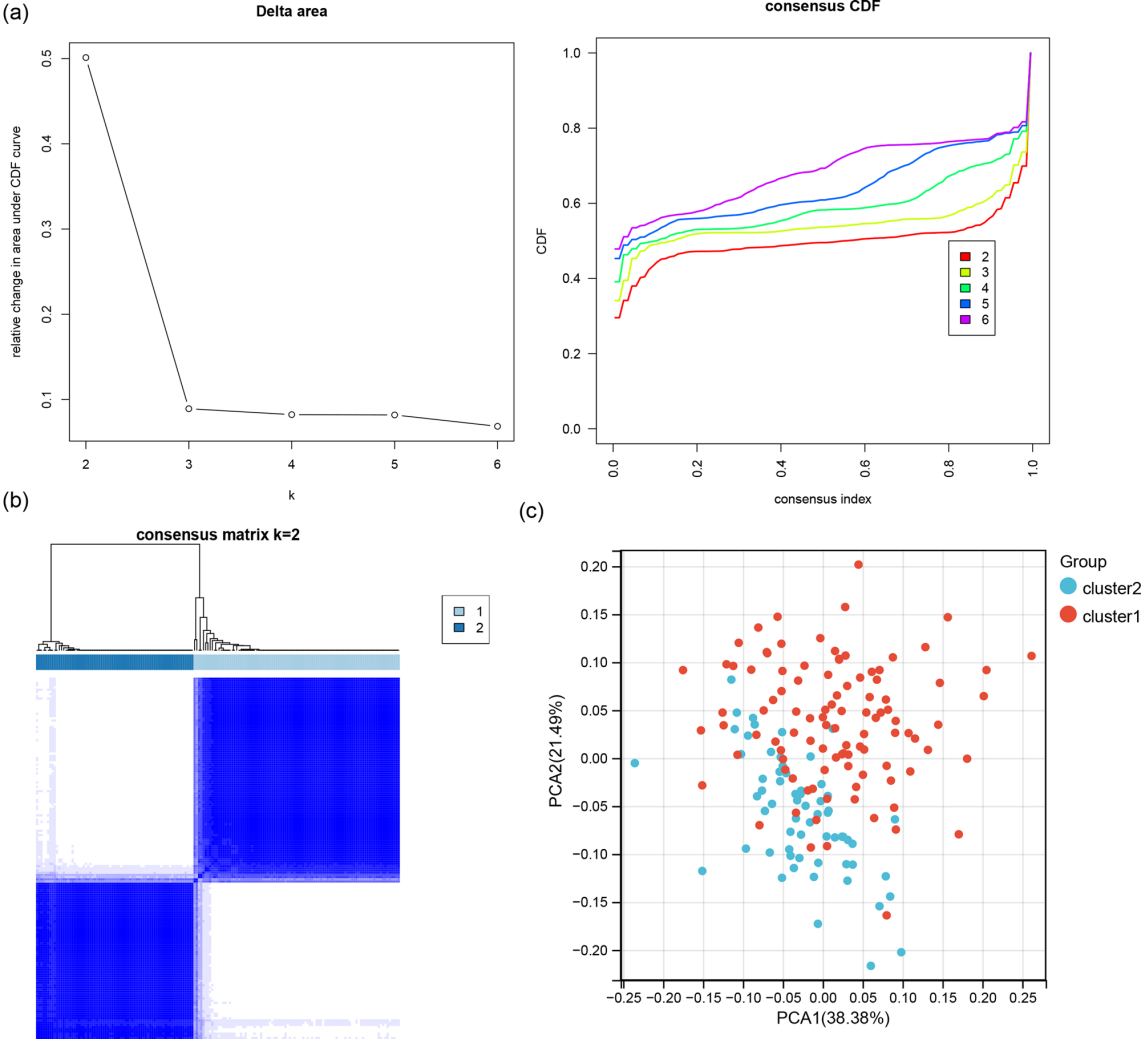
Consensus cluster analysis. (a) Parameter selection for consensus cluster analysis. (b) Clustering effect of two subtypes of samples. (c) PCA diagram of principal component analysis.

With the obtained expression matrix of intersection genes, consensus cluster analysis was conducted for gastric cancer samples. Combined with cumulative distribution function value, the best way of clustering was selected (Figure 3a), and two subtypes (*K* = 2) were identified (Figure 3b). PCA was used to evaluate the sample clustering results. As shown in Figure 3c, two subtypes of samples exhibit clear distribution patterns.

### 3.4 Survival analysis of patients in two subtypes

**Figure 4:**
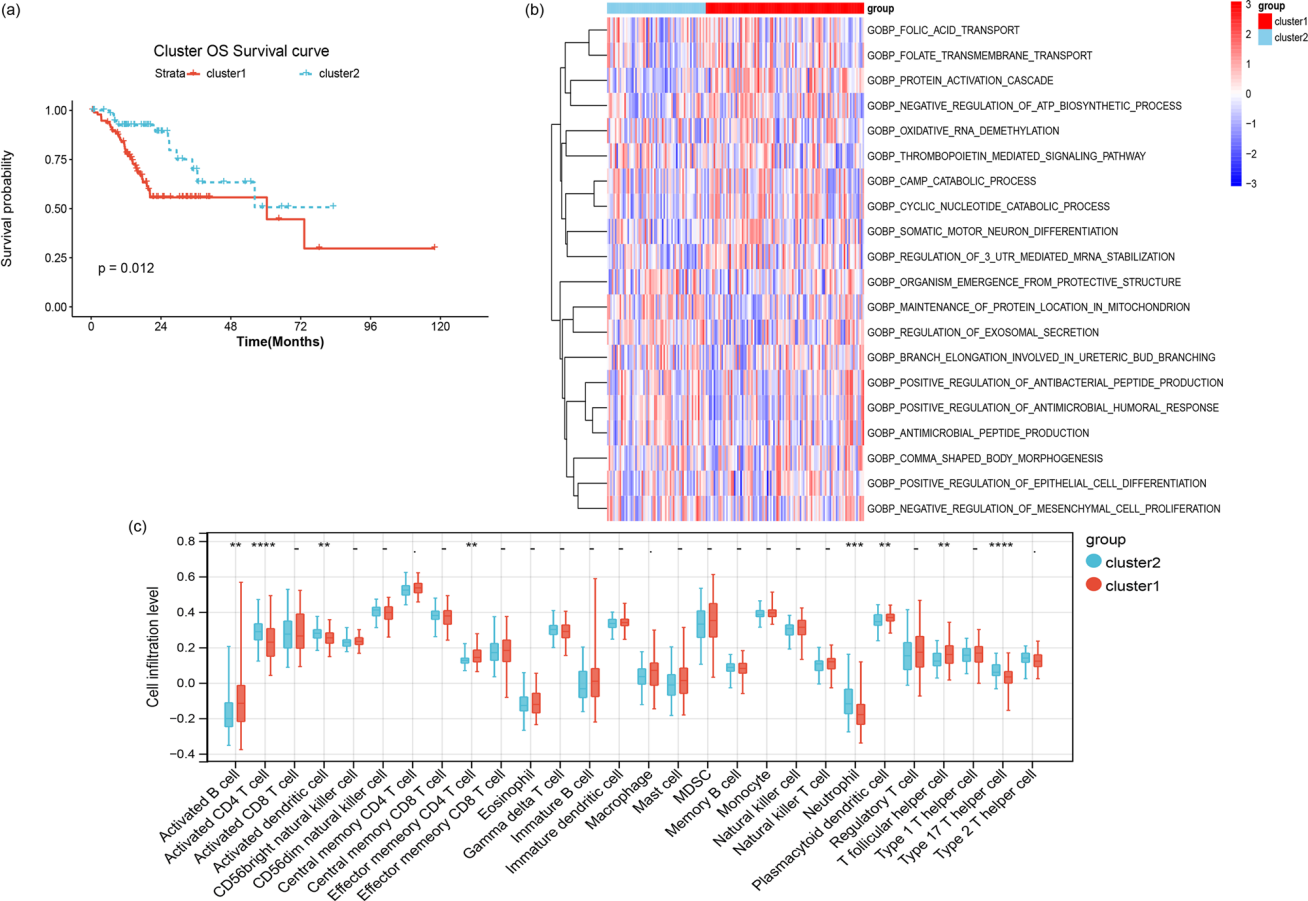
Difference analysis of patients in two subtypes. (a) KM graph of patients with different subtypes. (b) Enrichment heat map of TOP20 differential functional pathways among two subtypes. (c) Box plot of immune cell infiltration abundance between two subtypes. The difference of immune cell infiltration was analyzed between subgroups using wilcox.test.

To compare the survival differences of patients in two subtypes, KM curves for patients in two subtypes were drawn (Figure 4a). The results revealed significant survival differences for patients in different subtypes, and patients in cluster 2 had a better prognosis than in cluster 1 (*p* = 0.012).

### 3.5 Pathway analysis between two subtypes

According to the significance threshold *p* value <0.05 and |score| >2, 134 differential pathways were obtained, such as folic acid transport, protein activation cascade, oxidative RNA demethylation, and thrombopoietin-mediated signaling pathway. The heatmap of the differential enrichment pathways (top 20) is shown in Figure 4b.

### 3.6 Immune microenvironment analysis

Eight types of immune cells were differential in infiltration level between two subtypes, including activated B cell, activated CD4 T cell, activated dendritic cell, effector memory CD4 T cell, neutrophil, T follicular helper cell, plasmacytoid dendritic cell, and type 17T helper cell (Figure 4c).

### 3.7 Prognosis signature screening

**Figure 5:**
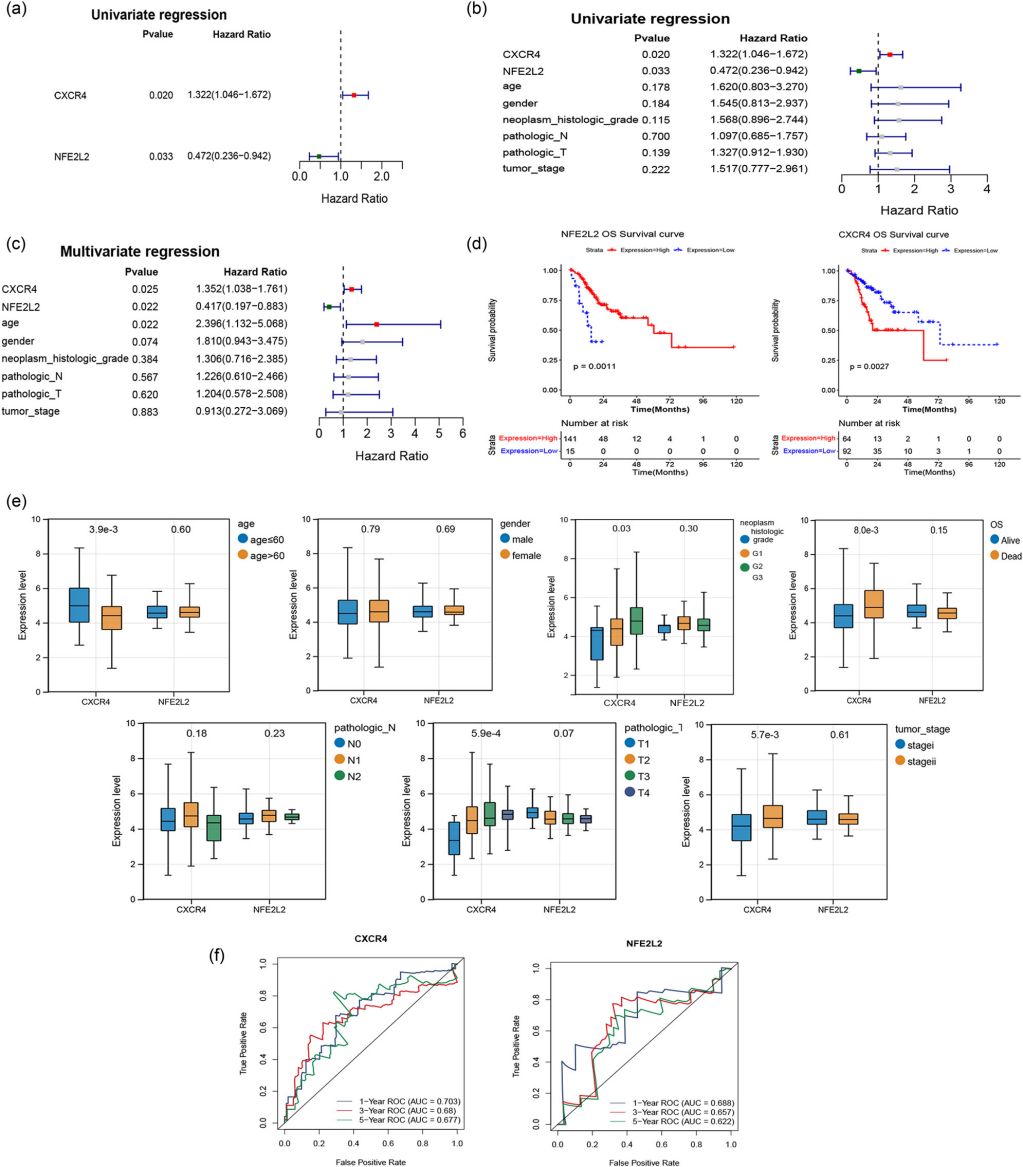
Selection of prognostic signatures. (a) Forest map of univariate Cox regression analysis. (b) Univariate independent prognostic analysis. (c) Multivariate independent prognostic analysis. (d) KM survival graph for two prognostic biomarkers. (e) Boxplots of prognostic biomarkers expression in different clinical features. The correlations between biomarkers and clinical features were analyzed by wilcox.test or Kruskal–Wallis method. (f) ROC analysis of the predictive value of CXCR4 and NFE2L2 for 1, 3, and 5-year survival rate in gastric cancer patients.

The univariate Cox proportional risk regression analysis was performed for 43 differentially expressed NRGs. With *p* value <0.05, two genes (*CXCR4* and *NFE2L2*) were selected (Figure 5a). Combined with the clinical variables, univariate and multivariate regression analyses were performed to evaluate the prognostic value of *CXCR4* and *NFE2L2*. Results indicated that *CXCR4* and *NFE2L2* were the independent risk factor for the prognosis of gastric cancer (*p* < 0.05, Figure 5b and c). Based on the two genes above, stage I and stage II gastric cancer samples were divided into high- and low-expression group. Then KM survival analysis was conducted for patients in two groups (Figure 5d). The results showed that two genes were significantly related to patient survival. Correlation analysis revealed that *CXCR4* showed significant differences in clinical features of age, neoplasm histologic grade, OS, pathologic T, and tumor stage (Figure 5e). *CXCR4* and *NFE2L2* showed good prognostic value with AUC > 0.6. *CXCR4* exerted better predictive performance than *NFE2L2* in 1-, 3- and 5-year survival rate (Figure 5f).

### 3.8 GSEA of single gene

**Figure 6:**
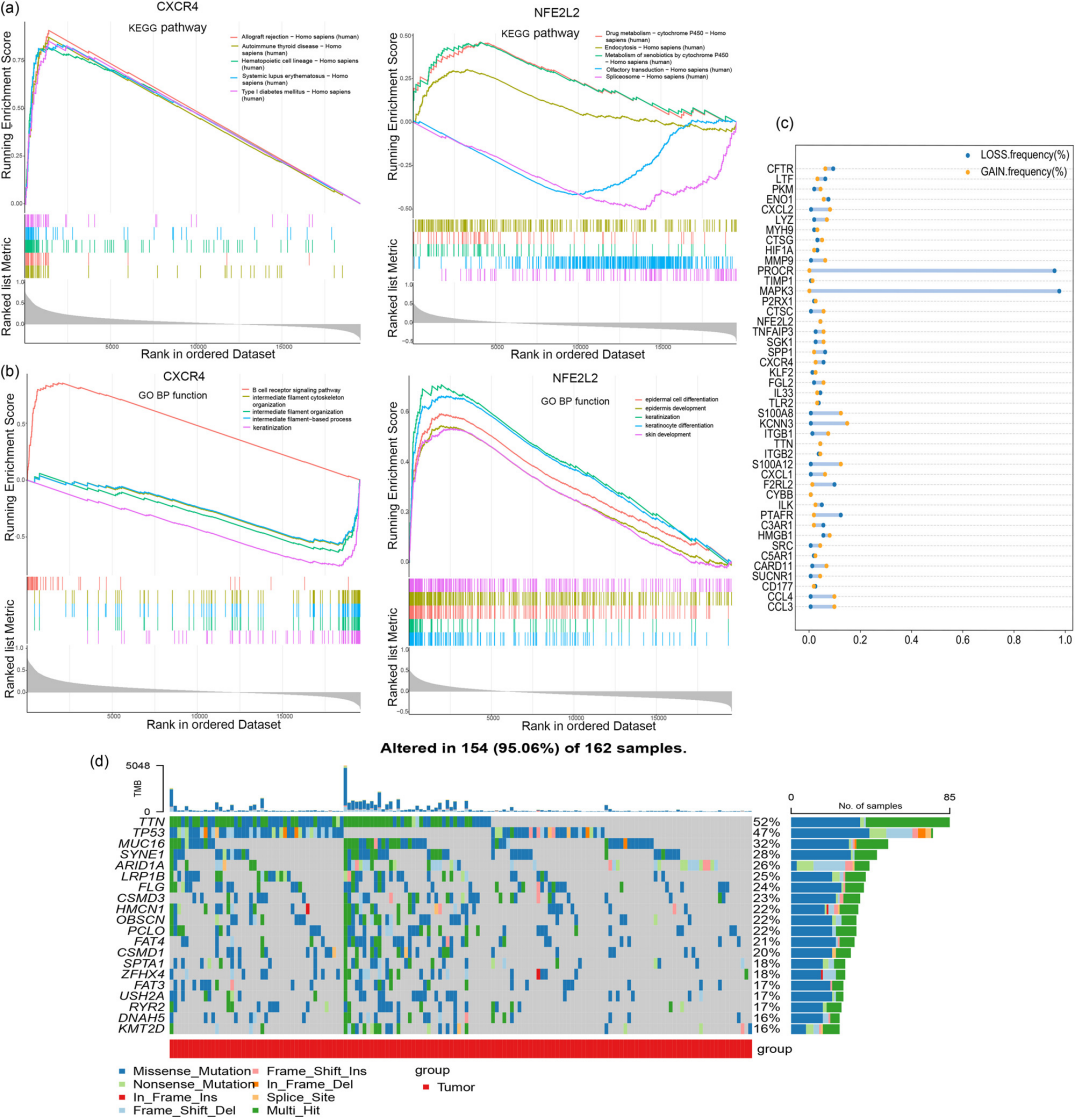
GSEA for CXCR4 and NFE2L2. (a) GO enrichment results (TOP5) of CXCR4 and NFE2L2. (b) KEGG enrichment results (TOP5) of CXCR4 and NFE2L2. (c) Gene mutation in stage I and stage II gastric cancer patients. (d) Copy number analysis of differentially expressed NETs.

The top five KEGG pathways for *CXCR4* were allograft rejection, hematopoietic cell lineage, autoimmune thyroid disease, systemic lupus erythematosus, and type I diabetes mellitus; for *NFE2L2* were drug metabolism-cytochrome P450, endocytosis, metabolism of xenobiotics by cytochrome P450, olfactory transduction, and spliceosome (Figure 6a). The top five GO terms for *CXCR4* were B cell receptor signaling pathway, intermediate filament organization, keratinization, etc.; for *NFE2L2* the significantly enriched GO-BP terms included epidermis development, epidermal cell differentiation, keratinization, etc. (Figure 6b).

### 3.9 SNV and CNV analysis

As illustrated in Figure 6c, the frequency of TTN gene mutation is highest in stage I and stage II gastric cancer. Among the differentially expressed NRGs, *PROCR* and *MAPK3* showed highest copy number loss frequency in stage I and stage II gastric cancer patients (Figure 6d).

### 3.10 Drug sensitivity analysis

**Figure 7:**
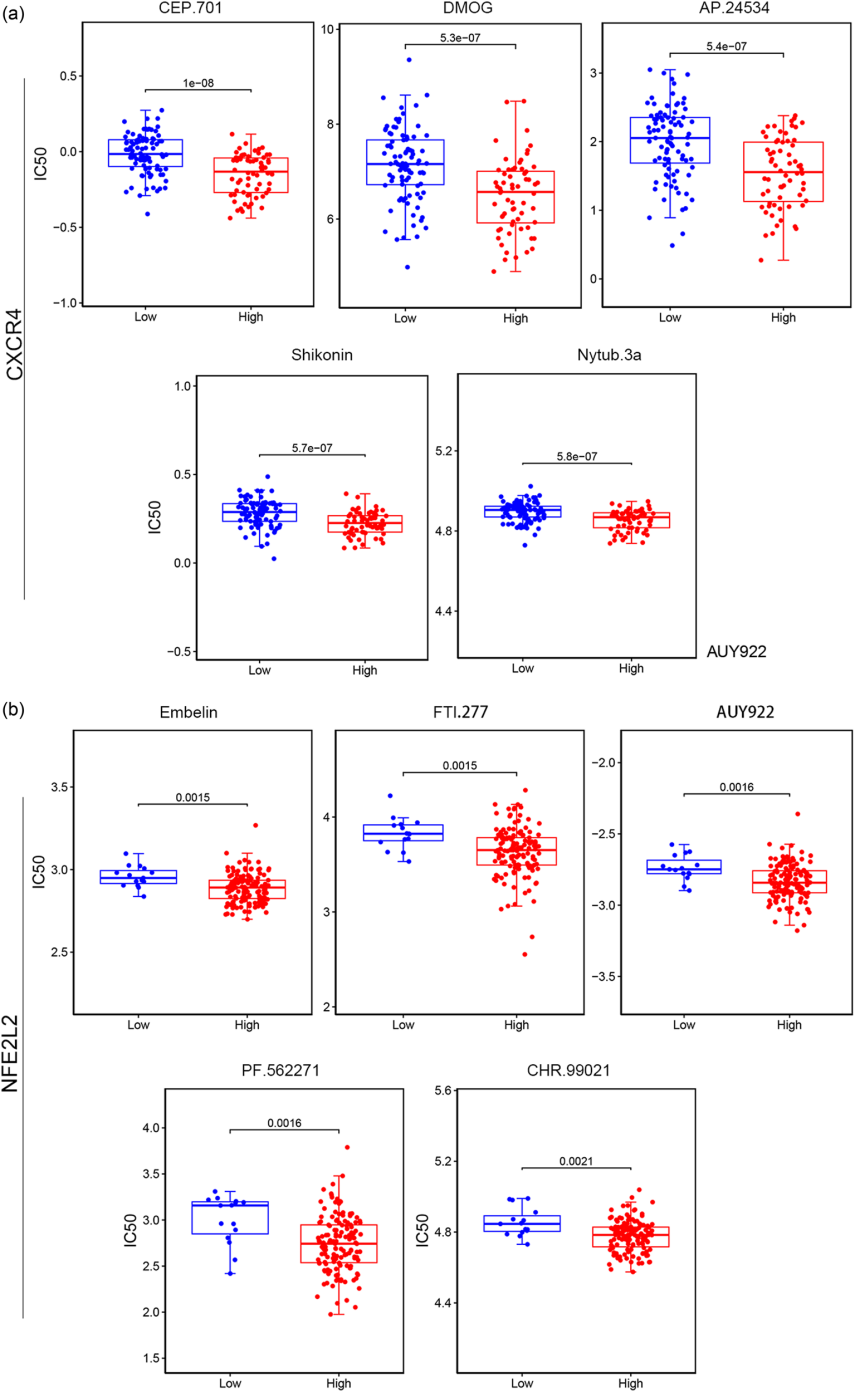
Drug sensitivity analysis. Box plot of the drugs with significant differences in sensitivity between high- and low-expression groups of e CXCR4 (a) and NFE2L (b). Statistically significant difference was analyzed by wilcox.test.

The drugs with significantly different sensitivity between high and low expression groups of each gene signature were analyzed by wilcox.test. The IC50 box plots of the top five drugs with significant differences in therapeutic sensitivity targeting *CXCR4* and *NFE2L2* are shown in Figure 7a and b.

### 3.11 Regulatory network analysis

**Figure 8:**
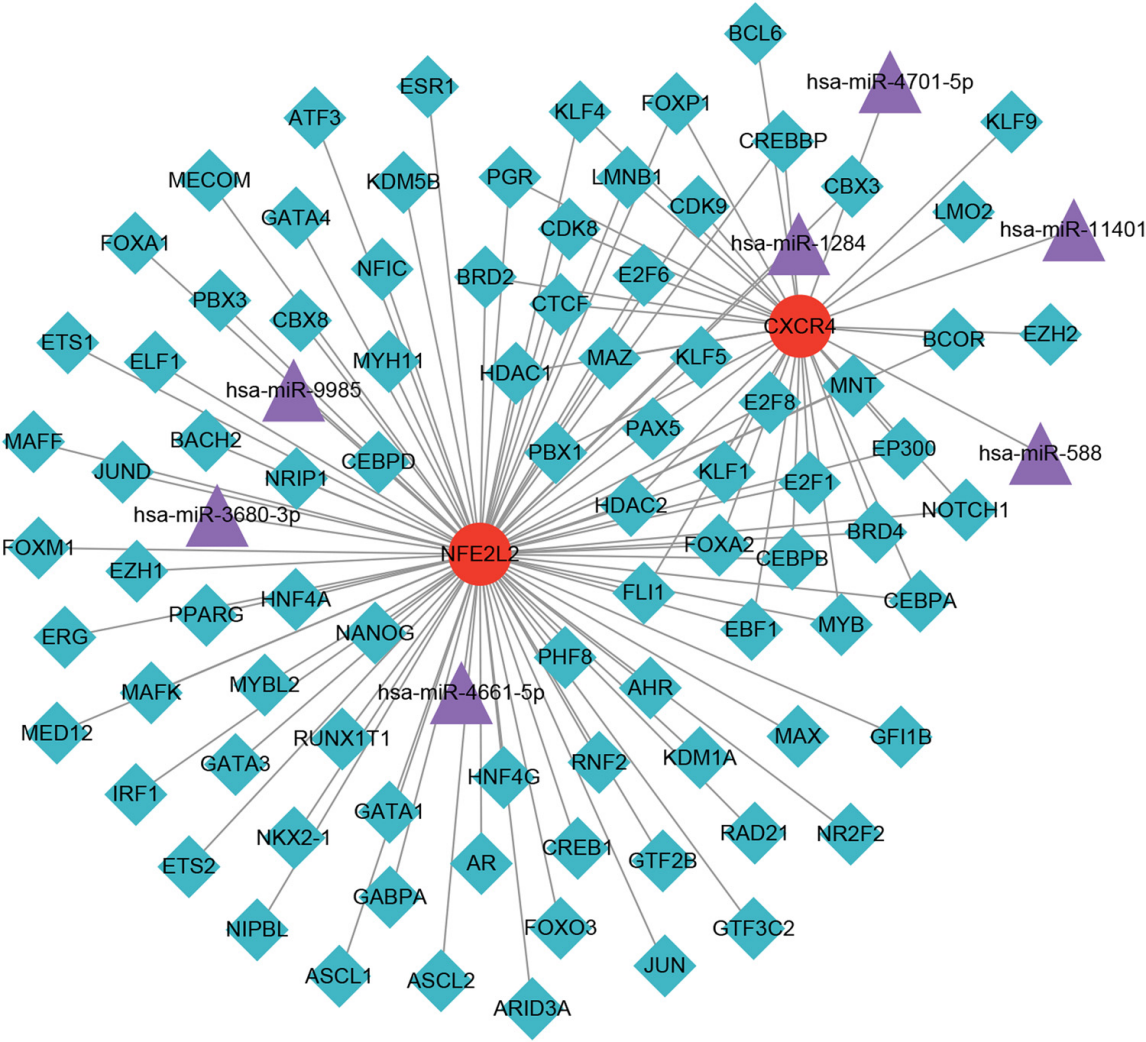
miRNA–mRNA–TF regulatory network diagram.

From miRwalk3.0 database, seven miRNA–mRNA pairs were predicted, involving seven miRNAs and two mRNAs. In hTFtarget database, 113 mRNA–TF pairs were predicted, involving 87 TFs and two mRNAs. The miRNA–mRNA pairs and mRNA–TF pairs obtained above were further screened for miRNAs and TFs regulated by the same mRNA. Finally, 421 miRNA–mRNA–TF regulatory pairs were identified and the network was visualized in Figure 8.

### 3.12 Validation of expression and prognostic value of gene signatures

**Figure 9:**
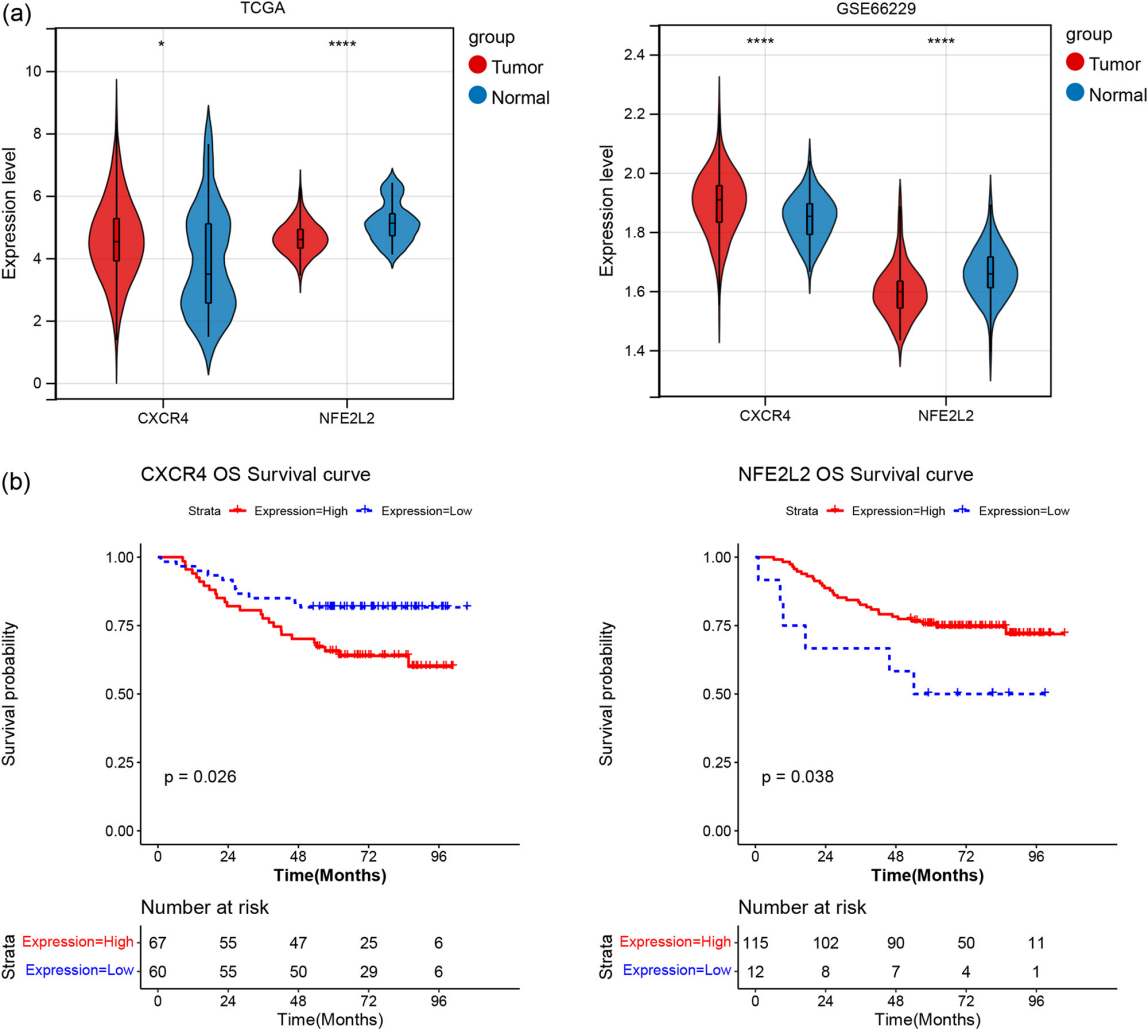
Validation of expression and prognostic value of two gene signatures. (a) Gene expression violin diagram of prognostic signatures. (b) KM survival graph of two gene signatures. The difference was analyzed by wilcox.test method. *p < 0.05, ****p < 0.0001, compared tumor group with normal group.

The expression levels of gene signatures were extracted from the training dataset (TCGA dataset) and the validation dataset (GSE66229). There were significant differences between the two gene signatures in both the training and the verification sets. CXCR4 showed an up-regulated trend and NFE2L2 showed a down-regulated trend in stage I and stage II gastric cancer samples (Figure 9a).

In addition, in GSE66229, stage I and stage II gastric cancer samples were also divided into high- and low-expression group. Combined with the survival information of patients, KM survival analysis also showed survival differences between the two groups, which were consistent with the training set (Figure 9b).

### 3.13 Validation of six key gene expression in cells and clinical samples

**Figure 10:**
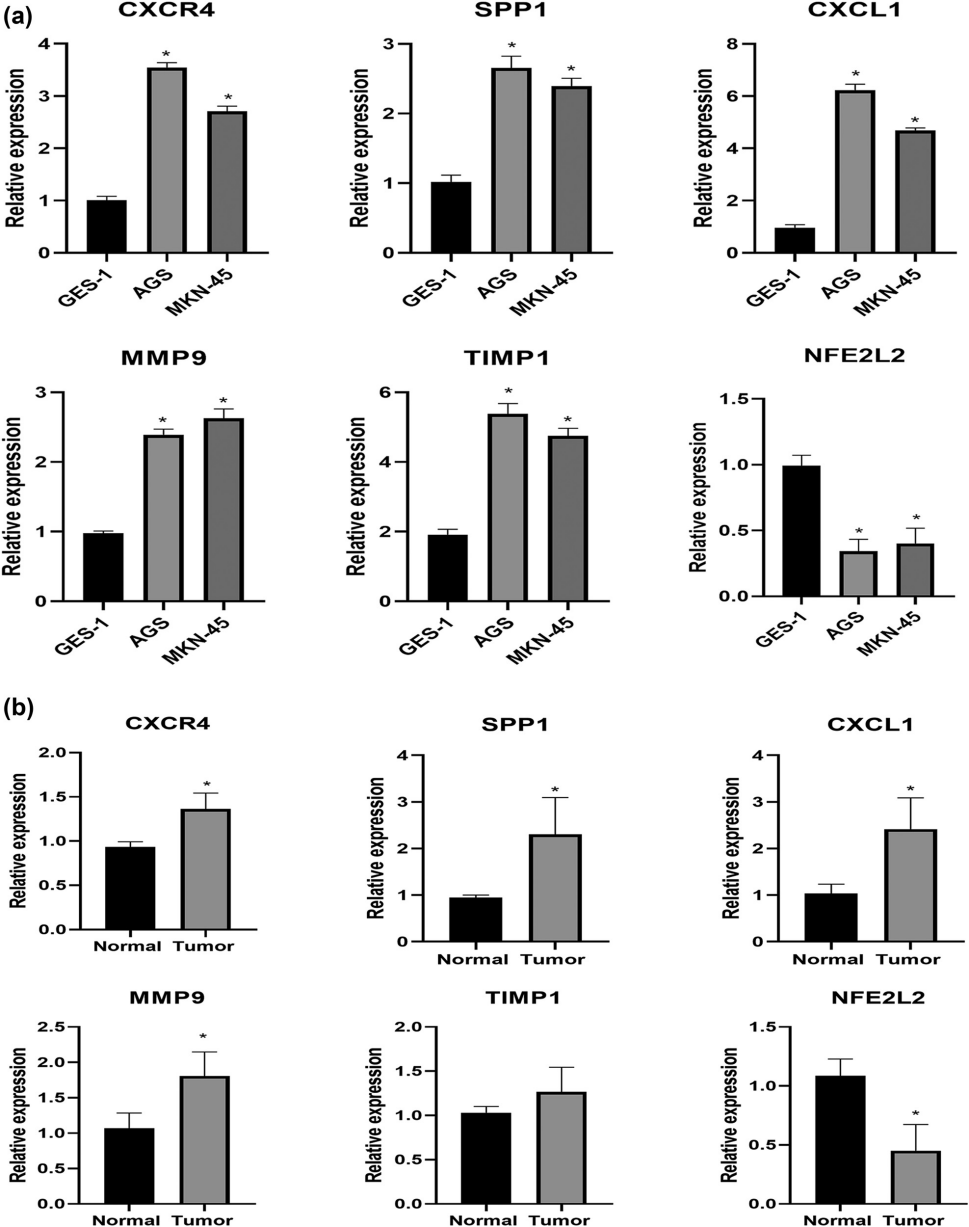
Validation of the expression of *CXCR4*, *NFE2L2*, *SPP1*, *CXCL1*, *MMP9*, and *TIMP1*. (a) qRT-PCR analysis in two gastric cancer cell lines. Human normal gastric mucous epithelium cell GES-1 was used as the control. (b) qRT-PCR analysis of gene expression in gastric tumor samples and normal controls. The comparison of gene expression between gastric cancer group and control group was performed using *t*-test. *p < 0.05, compared with control group.

The expression levels of *CXCR4* and *NFE2L2* as well as four differentially expressed NRGs (*SPP1*, *CXCL1*, *MMP9*, and *TIMP1*) were validated in gastric tumor cells and clinical samples. As shown in Figure 10a, *CXCR4*, *SPP1*, *CXCL1*, *MMP9*, and *TIMP1* were up-regulated and *NFE2L2* was down-regulated in gastric cancer cell lines compared with control. The results were consistent with the bioinformatic analysis. For clinical validation, no significant significance was found in *TIMP1* expression in tumor samples compared to normal ones (*p* > 0.05, Figure 10b), which could be due to the small size of samples. Further validation experiments are warranted.

## 4 Discussion

NETs play a key role in innate immune response [25], and study has suggested that NETs participate in various BP of human cancers [26]. Specially, NETs can facilitate tumor metastasis by catching the circulating cancer cells [27]. NETs have been reported to be associated with the clinical outcome in cancers [28]. The present study conducted a bioinformatic analysis regarding the NRGs in the subtypes and prognosis of stage I and stage II gastric cancer.

Consensus cluster analysis [29] is a common classification method of cancer subtypes, which can be used to classify samples into several subtypes according to different data sets, so as to discover new subtypes of diseases. Stage I and stage II gastric cancer samples were divided into two subtypes based on the NRGs. PCA revealed that the two subtypes of samples had clear distribution patterns, suggesting the reliability of the clustering result. Survival analysis revealed that patients in cluster 2 had a better prognosis compared to cluster 1. Thus, it is possible to predict the prognosis of patients by judging their subtypes.

Tumor microenvironment is a complex system composed of tumor cells, immune infiltrating cells, fibroblasts, cytokines, and catalytic factors [30]. Immune response plays an important role in tumor growth, metastasis, and invasion, thus tumor infiltrating immune cells could be the target of chemotherapy and radiation therapy. Immune cell infiltration in tumor is closely associated with the clinical outcome, and these infiltrated immune cells are the most likely drug targets to improve the survival rate of patients. As a result, we analyzed the immune cell infiltration in two subtypes, and found that eight types of immune cells were differential in infiltration level between two subtypes, among which four types were T cells. T cell response is necessary to control the tumors. During the development of cancer, T cells gradually become dysfunctional and exhaust [31]. Recent study found that T cell immunity affects the progress and prognosis of gastric cancer by participating in antitumor responses [32]. In addition to T cell, neutrophils were also differentially infiltrated between two clusters. Neutrophils have been reported to produce genotoxic substances, like reactive oxygen species, which could damage the epithelial cells DNA and trigger the action of tumors [33]. Thus, we speculated that these differential immune cells may serve as biomarkers or immunotherapy targets in patients of different subtypes.

Following consensus clustering analysis, univariate Cox proportional risk regression analysis was applied to screen prognostic signature, and *CXCR4* and *NFE2L2* were finally selected. *CXCR4* is expressed in a variety of epithelial cancer cells, and modulates the proliferation, survival, and migration of cancer cells [34]. Overexpression of *CXCR4* is significantly associated with lymphatic metastasis of gastric cancer [35]. Thus, it is not surprising that *CXCR4* showed significant differences in neoplasm histologic grade, OS, pathologic T, and tumor stage. GSEA of *CXCR4-*related genes showed that type I diabetes mellitus was a significantly enriched pathway. Much effect has been put to solve the relationship between diabetes mellitus and gastric cancer. The study of Sekikawa et al. has indicated that diabetes mellitus is a risk factor for early gastric cancer development [36]. A previous meta-analysis has revealed that diabetes mellitus shows a link with the incidence and mortality of gastric cancer [37]. The chemokine signaling mediated by *CXCR4* plays a key role in modulating the activity of myeloid-derived suppressor cells that had function in regulating insulin tolerance in diabetes [38]. *CXCR4* related signaling has been proposed as the promising therapeutic target of gastric cancer [39]. All these revealed the significant role of *CXCR4* in gastric cancer.

*NFE2L2* is the main regulator of cellular oxidative stress, which regulates the expression of some genes with cellular protective and antioxidant functions [40]. *NFE2L2* seems to have a dual role in cancer. Activation of the *NFE2L2* pathway is a potential preventive strategy against carcinogenesis [41]. Another study suggested that high *NFE2L2* expression is associated with aggressive tumor behavior [42]. Now its role in gastric cancer has not been fully investigated. GSEA revealed that *NFE2L2* was involved in two cytochrome P450-related pathways. Cytochrome P450 plays a critical role in exogenous metabolism of many potential carcinogens and anticancer drugs. It can inactivate or activate most carcinogens [43]. Hu and Chen [44] reported that P450 family genes associated with gastric cancer occurrence by stimulating the metabolism of exogenous anticancer drugs. It is reported that miRNAs that regulate *VEGFA* and *NFE2L2* could serve as the therapeutic targets in various cancers [45]. To investigate the regulatory mechanisms of *CXCR4* and *NFE2L2*, we constructed a miRNA–mRNA–TF regulatory network. *CXCR4* was regulated by three miRNAs (miR-588, miR-4701-5p, and miR-11401) and *NFE2L2* was regulated by miR-4661-5p, miR-1284, miR-3680-3p, and miR-9985. Among these miRNAs, miR-588 is involved in the progression of gastric cancer and down-regulation of miR-588 indicates a poor prognosis in gastric cancer [46]. miR-4661-5p is up-regulated in gastric cancer cells and down-regulated miR-4661-5p decreases the malignant behaviors of gastric cancer cells [47]. Wei et al. [48] demonstrated that miR-1284 suppressed the progression of gastric cancer by regulating *EIF4A1*. Furthermore, many TFs were predicted. Among these TFs, several regulated two genes at the same time. For instance, PAX5 is a functional tumor suppressor in gastric cancer and PAX5 promoter methylation is related to the survival of gastric cancer patients [49]. Down-regulated E2F6 in gastric adenocarcinoma is considered as an aggressive phenotype [50]. Taken together, these findings further suggested the critical roles of *CXCR4* and *NFE2L2* in predicting the prognosis of stage I and stage II gastric cancer patients. The exact regulatory mechanisms need further experimental validation.

In conclusion, the present study identified two subtypes and a prognostic signature for stage I and stage II gastric cancer based on NRGs, such as *CXCR4* and *NFE2L2*. This study may provide new evidence for prognosis prediction of patients with stage I and stage II gastric cancer.

## Supplementary Material

Supplementary Table

## References

[1] Sung H, Ferlay J, Siegel RL, Laversanne M, Soerjomataram I, Jemal A, et al. Global cancer statistics 2020: GLOBOCAN estimates of incidence and mortality worldwide for 36 cancers in 185 countries. CA A Cancer J Clin. 2021;71(3):209–49. 10.3322/caac.21660 33538338

[2] Bray F, Ferlay J, Soerjomataram I, Siegel RL, Torre LA, Jemal A. Global cancer statistics 2018: GLOBOCAN estimates of incidence and mortality worldwide for 36 cancers in 185 countries. CA A Cancer J Clin. 2018;68(6):394–424. 10.3322/caac.21492 30207593

[3] Reim D, Loos M, Vogl F, Novotny A, Schuster T, Langer R, et al. Prognostic implications of the seventh edition of the international union against cancer classification for patients with gastric cancer: the Western experience of patients treated in a single-center European institution. J Clin Oncol. 2013;31(2):263–71. 10.1200/JCO.2012.44.4315 23213098

[4] Fang T, Yin X, Wang Y, Zhang L, Zhang X, Zhao X, et al. Proposed models for prediction of mortality in stage-I and stage-II gastric cancer and 5 years after radical gastrectomy. J Oncol. 2022;2022:4510000. 10.1155/2022/4510000 PMC8923749 35300349

[5] Huang Z, Zhu D, Wu L, He M, Zhou X, Zhang L, et al. Six serum-based miRNAs as potential diagnostic biomarkers for gastric cancer. Cancer Epidemiol Biomark Prev. 2017;26(2):188–96. 10.1158/1055-9965.EPI-16-0607 27756776

[6] Chavakis T, Mitroulis I, Hajishengallis G. Hematopoietic progenitor cells as integrative hubs for adaptation to and fine-tuning of inflammation. Nat Immunol. 2019;20(7):802–11. 10.1038/s41590-019-0402-5 PMC6709414 31213716

[7] Jorch SK, Kubes P. An emerging role for neutrophil extracellular traps in noninfectious disease. Nat Med. 2017;23(3):279–87. 10.1038/nm.4294 28267716

[8] Yousefi S, Mihalache C, Kozlowski E, Schmid I, Simon H-U. Viable neutrophils release mitochondrial DNA to form neutrophil extracellular traps. Cell Death Differ. 2009;16(11):1438–44. 10.1038/cdd.2009.96 19609275

[9] Cristinziano L, Modestino L, Antonelli A, Marone G, Simon H-U, Varricchi G, et al. editors. Neutrophil extracellular traps in cancer. Seminars in cancer biology. Elsevier; 2022. 10.1016/j.semcancer.2021.07.011 34280576

[10] Rayes RF, Mouhanna JG, Nicolau I, Bourdeau F, Giannias B, Rousseau S, et al. Primary tumors induce neutrophil extracellular traps with targetable metastasis promoting effects. JCI Insight. 2019;5(16). 10.1172/jci.insight.128008 PMC6777835 31343990

[11] Wu J, Zhang F, Zheng X, Zhang J, Cao P, Sun Z, et al. Identification of renal ischemia reperfusion injury subtypes and predictive strategies for delayed graft function and graft survival based on neutrophil extracellular trap-related genes. Front Immunol. 2022;13:1047367. 10.3389/fimmu.2022.1047367 PMC9752097 36532016

[12] Smyth GK. Limma: linear models for microarray data. Bioinformatics and computational biology solutions using R and bioconductor. 2005. p. 397–420.

[13] Wickham H, Chang W, Wickham MH. Package ‘ggplot2’. Create elegant data visualisations using the grammer of graphics Version. 2016;2(1):1–189.

[14] Kolde R, Kolde MR. Package ‘pheatmap’. R package. 2018;1(10).

[15] Bardou P, Mariette J, Escudié F, Djemiel C, Klopp C. jvenn: an interactive Venn diagram viewer. BMC Bioinform. 2014;15(1):1–7. 10.1186/1471-2105-15-293 PMC4261873 25176396

[16] Zhang H, Meltzer P, Davis S. RCircos: an R package for Circos 2D track plots. BMC Bioinform. 2013;14:1–5. 10.1186/1471-2105-14-244 PMC3765848 23937229

[17] Wilkerson MD, Hayes DN. ConsensusClusterPlus: a class discovery tool with confidence assessments and item tracking. Bioinformatics. 2010;26(12):1572–3. 10.1093/bioinformatics/btq170 PMC2881355 20427518

[18] Wang P, Wang Y, Hang B, Zou X, Mao J-H. A novel gene expression-based prognostic scoring system to predict survival in gastric cancer. Oncotarget. 2016;7(34):55343. 10.18632/oncotarget.10533 PMC5342421 27419373

[19] Liberzon A, Subramanian A, Pinchback R, Thorvaldsdóttir H, Tamayo P, Mesirov JP. Molecular signatures database (MSigDB) 3.0. Bioinformatics. 2011;27(12):1739–40. 10.1093/bioinformatics/btr260 PMC3106198 21546393

[20] Hänzelmann S, Castelo R, Guinney J. GSVA: gene set variation analysis for microarray and RNA-seq data. BMC Bioinform. 2013;14:1–15. 10.1186/1471-2105-14-7 PMC3618321 23323831

[21] Kassambara A, Kosinski M, Biecek P, Fabian S. Package ‘survminer’. Drawing survival curves using ‘ggplot2’(R package version 03 1). 2017.

[22] Geeleher P, Cox N, Huang RS. pRRophetic: an R package for prediction of clinical chemotherapeutic response from tumor gene expression levels. PLoS One. 2014;9(9):e107468. 10.1371/journal.pone.0107468 PMC4167990 25229481

[23] Zhang Q, Liu W, Zhang H-M, Xie G-Y, Miao Y-R, Xia M, et al. hTFtarget: a comprehensive database for regulations of human transcription factors and their targets. Genom Proteom Bioinform. 2020;18(2):120–8. 10.1016/j.gpb.2019.09.006 PMC7647694 32858223

[24] Smoot ME, Ono K, Ruscheinski J, Wang P-L, Ideker T. Cytoscape 2.8: new features for data integration and network visualization. Bioinformatics. 2011;27(3):431–2. 10.1093/bioinformatics/btq675 PMC3031041 21149340

[25] Khan U, Chowdhury S, Billah MM, Islam KMD, Thorlacius H, Rahman M. Neutrophil extracellular traps in colorectal cancer progression and metastasis. Int J Mol Sci. 2021;22(14):7260. 10.3390/ijms22147260 PMC8307027 34298878

[26] Demkow U. Neutrophil extracellular traps (NETs) in cancer invasion, evasion and metastasis. Cancers. 2021;13(17):4495. 10.3390/cancers13174495 PMC8431228 34503307

[27] Masucci MT, Minopoli M, Del Vecchio S, Carriero MV. The emerging role of neutrophil extracellular traps (NETs) in tumor progression and metastasis. Front Immunol. 2020;11:1749. 10.3389/fimmu.2020.01749 PMC7524869 33042107

[28] Zhang Y, Guo L, Dai Q, Shang B, Xiao T, Di X, et al. A signature for pan-cancer prognosis based on neutrophil extracellular traps. J Immunother Cancer. 2022;10(6):2021-004210. 10.1136/jitc-2021-004210 PMC9189842 35688556

[29] Nguyen N, Caruana R, editors. Consensus clusterings. Seventh IEEE International Conference on Data Mining (ICDM 2007). IEEE; 2007.

[30] Anderson NM, Simon MC. The tumor microenvironment. Curr Biol. 2020;30(16):R921–5. 10.1016/j.cub.2020.06.081 PMC8194051 32810447

[31] Kamphorst AO, Ahmed R. CD4 T-cell immunotherapy for chronic viral infections and cancer. Immunotherapy. 2013;5(9):975–87. 10.2217/imt.13.91 PMC3864579 23998732

[32] Wei M, Shen D, Mulmi Shrestha S, Liu J, Zhang J, Yin Y. The progress of T cell immunity related to prognosis in gastric cancer. BioMed Res Int. 2018;2018. 10.1155/2018/3201940 PMC5848132 29682534

[33] Zhang X, Shi H, Yuan X, Jiang P, Qian H, Xu W. Tumor-derived exosomes induce N2 polarization of neutrophils to promote gastric cancer cell migration. Mol Cancer. 2018;17(1):1–16. 10.1186/s12943-018-0898-6 PMC6174070 30292233

[34] Burger JA, Kipps TJ. CXCR4: a key receptor in the crosstalk between tumor cells and their microenvironment. Blood. 2006;107(5):1761–7. 10.1182/blood-2005-08-3182 16269611

[35] Lee HJ, Kim SW, Kim HY, Li S, Yun HJ, Song KS, et al. Chemokine receptor CXCR4 expression, function, and clinical implications in gastric cancer. Int J Oncol. 2009;34(2):473–80. 19148483

[36] Sekikawa A, Fukui H, Maruo T, Tsumura T, Okabe Y, Osaki Y. Diabetes mellitus increases the risk of early gastric cancer development. Eur J Cancer. 2014;50(12):2065–71. 10.1016/j.ejca.2014.05.020 24934410

[37] Tian T, Zhang L, Ma X, Zhou J, Shen J. Diabetes mellitus and incidence and mortality of gastric cancer: a meta-analysis. Exp Clin Endocrinol Diabetes. 2011;217–23. 10.1055/s-0031-1297969 22187293

[38] Oya Y, Hayakawa Y, Koike K. Tumor microenvironment in gastric cancers. Cancer Sci. 2020;111(8):2696–707. 10.1111/cas.14521 PMC7419059 32519436

[39] Xue LJ, Mao XB, Ren LL, Chu XY. Inhibition of CXCL12/CXCR4 axis as a potential targeted therapy of advanced gastric carcinoma. Cancer Med. 2017;6(6):1424–36. 10.1002/cam4.1085 PMC5463074 28544785

[40] Karihtala P, Porvari K, Soini Y, Eskelinen M, Juvonen P, Haapasaari K-M. Expression levels of microRNAs miR-93 and miR-200a in pancreatic adenocarcinoma with special reference to differentiation and relapse-free survival. Oncology. 2019;96(3):164–70. 10.1159/000494274 30537722

[41] Kwak M-K, Kensler TW. Targeting NRF2 signaling for cancer chemoprevention. Toxicol Appl Pharmacol. 2010;244(1):66–76. 10.1016/j.taap.2009.08.028 PMC3584341 19732782

[42] Kawasaki Y, Okumura H, Uchikado Y, Kita Y, Sasaki K, Owaki T, et al. Nrf2 is useful for predicting the effect of chemoradiation therapy on esophageal squamous cell carcinoma. Ann Surg Oncol. 2014;21:2347–52. 10.1245/s10434-014-3600-2 24599410

[43] Ding X, Kaminsky LS. Human extrahepatic cytochromes P450: function in xenobiotic metabolism and tissue-selective chemical toxicity in the respiratory and gastrointestinal tracts. Annu Rev Pharmacol Toxicol. 2003;43(1):149–73. 10.1146/annurev.pharmtox.43.100901.140251 12171978

[44] Hu K, Chen F. Identification of significant pathways in gastric cancer based on protein-protein interaction networks and cluster analysis. Genet Mol Biol. 2012;35:701–8. 10.1590/S1415-47572012005000045 PMC3459423 23055812

[45] Cuzziol CI, Castanhole-Nunes MMU, Pavarino ÉC, Goloni-Bertollo EM. MicroRNAs as regulators of VEGFA and NFE2L2 in cancer. Gene. 2020;759:144994. 10.1016/j.gene.2020.144994 32721475

[46] Chen Y, Zhang J, Gong W, Dai W, Xu X, Xu S. miR-588 is a prognostic marker in gastric cancer. Aging (Albany NY). 2021;13(2):2101. 10.18632/aging.202212 PMC7880400 33323542

[47] Gu J, Chu K. Increased Mars2 expression upon microRNA‐4661‐5p‐mediated KDM5D downregulation is correlated with malignant degree of gastric cancer cells. Cell Biol Int. 2021;45(10):2118–28. 10.1002/cbin.11661 34273914

[48] Wei W, Cao W, Zhan Z, Yan L, Xie Y, Xiao Q. MiR-1284 suppresses gastric cancer progression by targeting EIF4A1. OncoTargets Ther. 2019;12:3965. 10.2147/OTT.S191015 PMC6535428 31190893

[49] Li X, Cheung K, Ma X, Tian L, Zhao J, Go M, et al. Epigenetic inactivation of paired box gene 5, a novel tumor suppressor gene, through direct upregulation of p53 is associated with prognosis in gastric cancer patients. Oncogene. 2012;31(29):3419–30. 10.1038/onc.2011.511 22105368

[50] Korourian A, Roudi R, Shariftabrizi A, Kalantari E, Sotoodeh K, Madjd Z. Differential role of Wnt signaling and base excision repair pathways in gastric adenocarcinoma aggressiveness. Clin Exp Med. 2017;17:505–17. 10.1007/s10238-016-0443-0 27909884

