# Supplementary material for: Identification and validation of molecular subtypes and prognostic signature for stage I and stage II gastric cancer based on neutrophil extracellular traps
Source: Open Med (Wars). 2024 Jan 12;19(1):20230860. doi: 10.1515/med-2023-0860 (PMC10787308; doi:10.1515/med-2023-0860)
Supplement: Supplementary Table [file med-2023-0860-sm.pdf]

Supplementary material

Table S1: Clinical characteristics of tumor patients and normal controls

Characteristics	Normal (*n* = 32)	Tumor (*n* 164)	*P* value
Age (years)			0.2177
< 60	5	45	
≥ 60	27	116	
Neoplasm_histologic_grade			0.813
G1	0	5	
G2	13	69	
G3	19	86	
Pathologic_M			0.02584
M0	28	154	
M1	2	0	
Pathologic_N			0.002379
N0	12	105	
N1	11	44	
N2	8	11	
Pathologic_T			0.5917
T1	3	18	
T2	11	63	
T3	13	70	
T4	5	13	
Gender			0.8069
Female	10	58	
Male	22	106	
Vital_status			1
Alive	23	115	
Dead	9	48	
Tumor_stage			1.00E-08
Stage i	6	53	
Stage ii	15	111	
Stage iii	6	0	
Stage iv	4	0	

Table S2: The primers used in this study

Gene symbol	Forward primer (5′→3′)	Reverse primer (5′→3′)
CXCR4	GGTCTGGAGACTATGACTCCA	GTGCTGGAACTGGAACACCA
NFE2L2	CCAGCACAACACATACCA	TAGCCGAAGAAACCTCATT
SPP1	GAAGTTTCGCAGACCTGACAT	GTATGCACCATTCAACTCCTCG
CXCL1	ATCTAGGAACCCCCTCCTCA	AGCATCCCTACCCTGCTGTA
MMP9	TTCCTTGGTCTGGTGTCCC	CCCACTTCTTGTCGCTGTC
TIMP1	AGACCTACACTGTTGGCTGTGAG	GACTGGAAGCCCTTTTCAGAG
